# Treating drug-resistant tuberculosis in a low-intensity chronic conflict setting in India

**DOI:** 10.1186/1752-1505-8-25

**Published:** 2014-12-01

**Authors:** Edward Armstrong, Mrinalini Das, Homa Mansoor, Ramesh B Babu, Petros Isaakidis

**Affiliations:** Médecins Sans Frontières, Chandni Bungalow, Union Park, Off Carter Road, Khar (W), Mumbai, 400 052 India; District TB Control Office (RNTCP), Khammam District, Andhra Pradesh India

**Keywords:** Operational research, Internally displaced populations, Resource-limited settings, Mobile clinic

## Abstract

**Introduction:**

The eastern part of India has been affected by an ongoing low-intensity conflict between government forces and armed Maoist groups, known as Naxalites. Since 2006, Médecins Sans Frontières (MSF) has been providing primary health care services in the conflict-affected region along the Andhra Pradesh-Chhattisgarh border. In 2011, treatment for drug-resistant tuberculosis (DR-TB) was included in the services provided. This report aims to describe MSF experiences of providing treatment to DR-TB patients in a mobile primary health care outpatient clinic, in a low-intensity conflict setting in India.

**Case description:**

A total of thirteen patients were diagnosed with drug-resistant TB (DR-TB) between January 2011 and October 2013. An innovative treatment model was developed which delegated responsibility to non-TB clinicians, including primary-care nurses and nurse-aids who were remotely supported by a TB-specialist from the MSF DR-TB project in Mumbai. Individualised regimens were designed for each patient based on WHO guidelines. Of these 13 patients, 10 patients had an outcome, of whom seven (70%) patients were cured. One patient became lost to follow-up prior to treatment initiation, one patient died prior to starting treatment and one patient refused treatment. Three patients were on-treatment, were clinically improving and were culture-negative at the end of their intensive phase of treatment.

**Discussion and evaluation:**

Drug-resistant tuberculosis diagnosis and treatment is a highly specialised and technical subject which requires continued patient follow-up. However, our study demonstrates that it is feasible to manage DR-TB patients in a conflict setting, using a primary-care model with remote expert support. Long-term commitment and sustainability are essential for continued care, even more so in similar conflict settings. Loss to follow-up in patients remains a programmatic challenge and community involvement may play a key role.

**Conclusion:**

Managing DR-TB in a primary health care programme is feasible in a low-conflict setting with an appropriate treatment model. Ambulatory strategies and standardised treatment regimens should be considered to further simplify treatment delivery and allow for scale-up when needed.

## Background

### The conflict

The eastern part of India has been affected by an ongoing low-intensity conflict between government forces and armed Maoist groups, known as Naxalites [1]. The regions primarily affected have heavily forested areas with significant unexploited natural resources, which contributes to an on-going armed power-struggle for access to these resources and their economic potential [2]. From 2005, around 6220 people have been killed in this conflict covering 12 states of Eastern India, with one third of these casualties in Chhattisgarh state alone [3]. The on-going low intensity conflict coupled with the significant displacement of the civilian population have resulted in poor access to health services [4].

### The health

The limited security and almost non-functional healthcare services in these conflict zones have hindered universal coverage of all national health programmes including tuberculosis (TB), and in particular drug-resistant tuberculosis (DR-TB). Multidrug-resistant tuberculosis (MDR-TB) is estimated to account for 2.1% of new cases and 15% of previously treated TB cases [5]. Reports from several states suggest that the prevalence of DR-TB may vary from 7% to over 50% [6–10]. The Revised National Tuberculosis Control Programme (RNTCP) has helped to enable MDR-TB patients access appropriate medication through government nationwide health structures [11, 12] however, coverage in conflict zones remains a challenge.

In 2006, in response to the conflict and the resultant significant civilian displacement to temporary relief camps Médecins Sans Frontières (MSF) started a primary health care mobile clinic service in the border area between the states of Chhattisgarh and Andhra Pradesh. This conflict may have contributed to the spread of DR-TB in the area by disrupting health care services, inhibiting both access of directly observed treatment (DOT) providers to their patients and also patient’s access to health services, interrupting drug supplies and the inevitable close habitation of DR-TB patients in temporary housing due to forced displacements. Thus, along with primary healthcare services, the MSF programme provided treatment to patients suffering from drug-sensitive TB and DR-TB.

Treatment of DR-TB is highly specialised, complex and expensive, and needs appropriate tools to monitor the long and painful regimen. Availability of these pre-requisites for DR-TB treatment and care in remote rural settings is uncertain. To date, little is known about DR-TB management in a primary health care setting in areas affected by conflict [13]. To address this knowledge gap, this report aims to describe MSF experiences of providing treatment to DR-TB patients in a mobile primary health care outpatient clinic, in a low-intensity conflict setting in India. We describe the treatment model that was followed in the MSF programme, particularly the use of a nurse-aid-implemented, remote-expert-guided approach to management. To our knowledge, this is the first report describing a treatment model providing DR-TB services to patients in a conflict setting in India [14, 15].

## Case description

### Setting and study population

Since 2006, MSF has been operational in the border region between Andhra Pradesh and Chhattisgarh (AP-CG). The project runs from the town of Bhadrachalam and has sub-bases in the small border towns of Konta and Cherla. The MSF mobile clinics provide care free of charge to patients and can help to facilitate referral to secondary care for patients requiring emergency care. The great majority of patients are from rural forested tribal areas often lacking in basic water or sanitation services or access to functioning healthcare facilities. In general, about one-third of patients attending the MSF clinics walk more than 10 km to reach the clinic location. MSF started providing DR-TB treatment to patients from January 2011. DR-TB care and treatment is offered in an outpatient primary healthcare setting.

The study population consisted of all DR-TB patients registered for care within the MSF programme between January 2011 and October 2013.

### Diagnostic and treatment model

All the patients presenting with presumed TB undergo sputum smear evaluation [16] in the Bhadrachalam designated microscopy centre (DMC) in Andhra Pradesh. A passive, facility-based case finding strategy is followed to identify patients with presumed TB. After smear evaluation, the following criteria are used to determine which TB patients are most at risk of having DR-TB.

- ▪ Category 1 and Category 2 treatment failure
- ▪ All retreatment cases and all cases starting Category 2 treatment
- ▪ Close contacts of known DR-TB patients
- ▪ HIV-infected TB patients

Patients who fall into the above categories are presumed to have a higher likelihood of DR-TB and their samples are then sent for sputum culture and drug susceptibility testing (DST). The following diagnostic and treatment model is being used:

1. Sputum collection and transfer to specialist laboratory for culture and Drug Susceptibility Testing (CDST): Two sputum samples, usually a spot and a morning sample, are collected and transferred via courier to the MSF HIV MDR-TB project in Mumbai, India. Delivery of these samples takes 2 working days and the sample is delivered to an RNTCP accredited private laboratory for culture and DST. Information regarding the possible DR-TB patient is made anonymous , entered into a database and sent via email to the MSF Mumbai MDR-TB technical referent. Since July 2013, the samples for line probe assay (LPA) are sent to Hyderabad RNTCP accredited laboratory, Andhra Pradesh.
2. A shared database (electronic- excel based) between the project staff and Mumbai TB technical referent is used to record the patients’ treatment progress. The database specifically monitors patients’ follow up sputum sample outcomes, blood test results and adverse events.
3. Pre-treatment evaluation: Following confirmation of a positive DR-TB result, a pre-treatment evaluation is carried out including basic hematological and biochemical investigations (including creatinine clearance) and an HIV test. Adherence counseling and health education sessions are given. A treatment consent form including a commitment to complete treatment is received from all the patients.
4. Individualised treatment regimen: The treatment protocols follow WHO guidelines [16]. However, during the initial years of the project, other international guidelines were followed [17]. Whenever possible, an individualized treatment regimen is designed for each patient by remote technical experts from the MSF Mumbai project. The treatment regimen is based on the first and second line DST results and on patients’ previous treatment histories. All TB drugs are dosed according to bodyweight. Dosing and drugs are also changed in response to severe adverse effects. Adverse events are aggressively managed and regimen modification is done as last resort if required. Medications are purchased and supplied by the MSF Mumbai project.
5. Treatment initiation and follow up: After treatment initiation, sputum smear and culture is repeated monthly until the end of the intensive treatment phase and every other month during the continuation phase. Chest X-ray is not routinely used for treatment monitoring. A directly-observed treatment (DOT) approach is followed depending on the level of insecurity in the area, using MSF community health workers, Furthermore, an MSF nurse, nurse aid or patient support officer would review the patient on a weekly basis to confirm compliance and report adverse events. If the patient remains stable, medical reviews by the MSF doctor occurs on a monthly basis. All treatment is provided free of charge. Additional supportive counseling is provided to patients who interrupt treatment. The tracing of lost-to-follow-up patients is carried out by a local team of MSF health workers.

### Data collection and analysis

Demographic and clinical information were recorded in patient files. The clinical data being routinely collected for each patient, including treatment and laboratory data, were entered into an electronic shared database. Data from all TB patients initiated on treatment between January 2011 and October 2013 were included in the analysis.

### Ethics

This case-study satisfied the criteria for reports using routinely collected programmatic data set by the Médecins Sans Frontières Ethics Review Board (ERB), Geneva, Switzerland. Patient identifying information was removed prior to analysis. As this was a study of routinely collected monitoring data, patient consent was not required.

### Patient treatment outcomes

Between Jan 2011 and October 2013, 13 patients were diagnosed with DR-TB (Table 1). The majority (80%) of them were males. All except one of the patients were adults. Six of the patients were from Andhra Pradesh and seven were from Chhattisgarh. None of the patients were found to be HIV-infected. All patients had pulmonary DR-TB: four patients had mono-drug resistant, four had poly-drug resistant and five had multidrug-resistant tuberculosis.

**Table 1:** **Clinical characteristics and treatment outcome details of pulmonary, drug-resistant TB patients in Chhattisgarh-Andhra Pradesh border, India**

Case	Age	Sex	State of origin	AFB smear result	Resistance profile DST	TB Resistance pattern	Treatment regimen	Duration (months)	Adverse events	Outcome
1	50	M	AP	Pos	S,H,Z	PDR	Inj Km/Mx/ R/E	14	Oto-toxicity	Cured
2	40	F	AP	Neg	H	Mono-R	Inj Km/Mx/ R/E/Z	14	-	Cured
3	25	M	AP	Neg	H,Z	PDR	-	-	-	LTFU
4	45	M	AP	Pos	S,H,R,Z,E	MDR	Inj Cm/Mx/ Eto/Cs/PAS	24	GI dist^a^	Cured
5	40	M	CG	Pos	H	Mono-R	Inj Cm/Mx/ R/E/Z	12	Psych^b^	Cured
6	45	M	CG	Pos	S,H,R,Z	MDR	Inj Cm/Mx/ Eto/Cs/PAS	24	-	Cured
7	45	M	CG	Pos	S,H,Eto	PDR	Inj Cm/Mx/ R/E/Z	13	Renal dys^c^	Cured
8	45	M	CG	Pos	S,H	PDR	Inj Cm/Mx/ R/E/Z	13	-	Cured
9	45	F	CG	Pos	H,R	MDR	Inj Km/Lx/ Eto/Cs/E/Z	7	-	On-treatment^┼^
10	13	F	CG	Pos	H	Mono-R	H/R/Z/E	6	-	On-treatment^┼^
11	35	M	AP	Neg	H,R,Z	MDR	Inj Km/Lx/ Eto/Cs/E/Z	7	-	On-treatment^┼^
12	25	M	CG	Pos	H,R	MDR	-	-	-	Died
13	60	M	AP	Pos	H	Mono-R	-	-	-	Refused treatment

M: Male, F: Female, AP: Andhra-Pradesh, CG: Chhattisgarh, Pos: Positive, Neg: Negative.

^a^GI dist: Gastro-intestinal disturbance, ^b^Psych: Psychological complaints, ^c^Renal dys : Renal dysfunction.

Mono-R: Mono-resistance tuberculosis; defined as drug-resistant TB case whose recovered M. tuberculosis isolate is resistant to one first line anti-TB drug.

PDR: Poly-resistance tuberculosis; defined as drug-resistant TB case whose recovered M. tuberculosis isolate is resistant to more than one first line anti-TB drug, but not MDR-TB.

MDR: Multi-drug resistant tuberculosis; defined as drug-resistant TB case whose recovered M. tuberculosis isolate is resistant in-vitro to isoniazid and rifampicin with or without resistance to other anti-tubercular drugs based on DST results.

S: Streptomycin, H: Isoniazid, R: Rifampicin, Z: Pyrazinamide, E: Ethambutol, Eto: Ethionamide, Km: Kanamycin, Mx: Moxifloxacin, Cm: Capreomycin, Cs: Cycloserine, PAS: Para-amino salicylic acid, Lx: Levofloxacin.

LTFU: Lost to follow up.

^┼^Culture negative at end of intensive phase; currently in continuation phase.

Of the thirteen patients initiated on treatment, seven patients completed treatment and were declared cured. Three patients were on-treatment, clinically improving and were culture-negative at the end of their intensive phase. The treatment regimen and duration for each patient is described in the table. One patient was lost to follow-up prior treatment initiation, one refused treatment and one died before starting treatment. In all, seven (70%) of ten patients with treatment outcome were cured. Adverse events were common (31%) among this cohort of patients. Among them, a patient had baseline renal dysfunction with a creatinine clearance of 21 ml/min, thus the treatment doses were adjusted in line with published recommendations. Another patient developed psychological disturbances after three months of treatment and required referral to a psychiatrist. The patients suffering from ototoxicity or gastrointestinal disturbances were managed symptomatically.

## Discussion and evaluation

Our experiences demonstrate that it is feasible to provide treatment to DR-TB patients within this particular conflict setting. The MSF position as a neutral actor, has allowed it to build trust and ensure continued access to the people it treats, in this chronic low-intensity conflict zone. On a day to day basis there is normally no immediate threat to the health workers which is vital for being able to deliver DR-TB medication on a daily basis. The patient cohort, rather than being direct victims of warfare find themselves as indirect victims. They are affected by intimidation, difficulties in travel and access to healthcare services due to the background security situation, as well as seasonal problems including monsoon rains and flooding. Our study shows that despite these challenges it is possible to deliver care and medications for the duration of the treatment period for DR-TB.

Our treatment model shows it is possible to manage and follow-up DR-TB patients in a primary care setting with non-specialists, rather than requiring a vertical DR-TB programme, if there is a system in place for supportive guidance from DR-TB experts, as previously documented in other MSF programmes [13]. It may be possible to adopt this approach in similar remote or conflict affected areas. In addition, as the expert guidance is provided remotely, it could be possible to create a network of decentralised primary healthcare providers who would use this same distance approach for the management of their DR-TB patients.

Our DR-TB patient cohort included similar number of patients from Chhattisgarh and Andhra Pradesh. This accurately reflects the border locations of the MSF clinics. About 23% (3/13) of the patients were women. Further, 7.7% (1/13) patients were children (<16 years old), which may reflect the ongoing challenges in diagnosing and managing children with TB [18]. However, limited conclusions on epidemiological profile of DR-TB patients may be drawn, due to the small size of the cohort.

There is little published evidence of the non-governmental organization (NGO) sector managing DR-TB in conflict settings. There is justifiable concern regarding trying to make a diagnosis if the expensive medications and any follow up system is not in place or is unrealistic given the context. Indeed, a sporadic or non-sustainable approach to DR-TB management in conflict settings could do more harm than good including leading to the amplification of resistance. Previous studies from Peru [19] and Mumbai [6] have demonstrated that it is possible to manage DR-TB patients in an ambulatory way without the need for in-patient services and this opportunity should be considered by other actors considering DR-TB programmes in conflict or remote settings.

A limitation to our treatment model is the varied treatment regimens used to treat our mono-resistant and poly-resistant DR-TB cohort, which in the current era may seem inappropriate. However, these regimens were prepared based on available literature and international guidelines then [16, 17]. The patients registered for care more recently were provided treatment based on new international [20] and national guidelines [21] published in recent years.

A second limitation was the prolonged duration of treatment for several of the patients. These were mostly due to delays in receiving patient laboratory reports or due to the patient’s severe or non-improving clinical condition. Another reason behind prolonged treatment was treatment interruptions by the patients. These patients missed appointments ranging from one week-one month during their treatment duration. The MSF staff had to trace back these patients by visiting their residence or sending confidential messages via residents from the same villages.

A few months after the MSF project in AP-CG region had been operational; the national TB programme (RNTCP) acknowledged the DR-TB burden in India and initiated the upscaling and investment in services for DR-TB. This includes the investment in molecular resistance testing including line probe assay (LPA) techniques and the availability of free DR-TB medications through the RNTCP. This enabled MSF to adapt its strategy in 2013 from one of being the provider of the entire DR-TB package of care for its patients and thus requiring a minimum commitment of two years in order to ensure the availability of medication for the duration of treatment and taking on more of a facilitator role. This has meant that new suspected DR-TB patients attending the MSF clinics may now have their diagnosis performed in one of the RNTCP accredited laboratories, their medications provided by the national programme but their clinical follow up and delivery of medications to the patients still conducted by the MSF mobile clinic staff. In this way, there is the reassurance that patients can in future be handed over to the RNTCP programme should the need arise albeit within the context of the challenging security situation.

Due to sufficient resources and small patient numbers, the DR-TB patients received an individualised treatment regimen based on standard treatment guidelines [16, 17] suggested by remote technical experts from the MSF Mumbai project. Whether this individualised approach is necessary for other similar projects operating in challenging contexts is questionable. If reliable DST is available to second line TB medications then the patient receive optimum treatment regimens however, in scenarios where there is significant cost or delay to achieving second line DST, or where patient numbers are sufficiently high to make individualised regimens too complex to manage, it may be appropriate to have a standardised DR-TB regimen which is used for all patients. We hypothesise that populations in contexts similar to ours may have low exposure to second-line TB medication, further justifying the need for standardised regimen. Actors designing a DR-TB programme should consider the local population resistance patterns and the feasibility, benefits and limitations of each option and take into account the scalability of each approach.

One problem encountered is regarding the patients who are lost to follow up either on a temporary basis or permanent, as was the case for one study patient. Significant difficulties exist regarding tracing patients who do not attend appointments or who are not available when their DOT provider goes to observe treatment. To try and limit missed doses, attempts are made to communicate in advance when patients needed to come to clinic, including sending reminders through respected members of the relevant villages. Furthermore, as a considerable number of patient villages are impossible to visit due to limited security, patients are strongly encouraged to come together with their close family members in order to encourage family support of the patient, thereby improving the adherence of the patients during treatment duration. The role of incentives [22] such as dry rations or cooked meals for patients during each visit should be explored to improve adherence.

## Conclusions

The drug-resistant tuberculosis diagnosis and treatment remains a highly specialised and technical subject which requires continued patient follow-up. However, in areas affected by low-intensity conflicts, our study has shown that with an appropriate treatment model and remotely given expert support, it is possible to implement a DR-TB programme within a primary health care project. The implementation of any such project requires a sustained commitment and an understanding of the implementation challenges.
